# Anaplastic Large-Cell Lymphoma (ALCL) First Manifested as a Rapidly Progressive Acute Respiratory Failure (RF): Lymph Node Biopsy Negative Presentation Posing a Diagnostic Challenge

**DOI:** 10.7759/cureus.37619

**Published:** 2023-04-15

**Authors:** Prabasha Weeraddana, Niwanthi Weerasooriya, Ragaa Elkabbani, Mohamed Zakee Mohamed Jiffry, Nepal Nisha, Manbir K Sandhu

**Affiliations:** 1 Internal Medicine, Danbury Hospital, Danbury, USA; 2 Pathology, Danbury Hospital, Danbury, USA

**Keywords:** lymph node-negative, negative bone marrow biopsy, patent bronchus, hilar lymphadenopathy, non-hodgkin lymphoma (nhl), anaplastic lymphoma kinase(alk), acute hypoxic respiratory failure, anaplastic large-cell lymphoma

## Abstract

Anaplastic large-cell lymphoma (ALCL) is an aggressive subtype of non-Hodgkin lymphoma. There are two forms of ALCL: primary and secondary. Primary can be systemic, affecting multiple organs, or cutaneous, affecting mainly the skin. A secondary form occurs when another lymphoma undergoes an anaplastic transformation. ALCL rarely presents as initial symptoms of respiratory failure. In most of these situations, the trachea or bronchial involved with an obstruction was present. We present an unusual case of ALCL where the patient rapidly progressed to acute hypoxic respiratory failure with a patent bronchus and trachea. Unfortunately, the patient rapidly deteriorated and died before diagnosis. Only upon at autopsy, it was found that his lung parenchyma was diffusely involved with ALCL. The autopsy report showed that the patient had CD-30 anaplastic lymphoma kinase (ALK)-negative ALCL diffusely involving all lung fields.

## Introduction

Anaplastic large-cell lymphoma (ALCL) is a non-Hodgkin lymphoma and one of the subtypes of T-cell lymphoma. It has a male predominance and is characterized by large, pleomorphic lymphoid cells [1]. It can affect various organs or just the skin. It can be anaplastic lymphoma kinase (ALK)-positive or negative. Generally present treatment approach for ALCL closely follows that of peripheral T-cell lymphomas (PTCL), in general. The most important risk factors include HIV infection and breast implants. We present a case of a 72-year-old male who presented with an episode of syncope and dyspnea and later rapidly progressed to acute hypoxic respiratory failure. It is an unusual case as the patient presented without any bronchial and tracheal obstruction, which is a common manifestation of lung involvement with respiratory failure in ALCL. Lymph node and bone marrow biopsies were negative which posed diagnostic challenges. The computerized tomography (CT) showed diffuse reticulonodular opacities with mediastinal and hilar lymphadenopathy. The patient developed shortness of breath on the second day of admission and was transferred to the ICU for further management. He was found to have thrombocytopenia and lymphocytosis. However, the patient’s condition worsened before the open lung biopsy and he passed away on the 18th day of hospitalization. 

## Case presentation

A 72-year-old male was presented to the emergency department following a syncopal episode at his primary care physician's office. He also complained of lightheadedness and dyspnea. The patient had a past medical history of coronary artery disease, emphysema, and chronic kidney disease (CKD) stage III. He is a current smoker with more than 80 pack years. He had been admitted a week prior for acute hypoxic respiratory failure due to multifocal pneumonia and suspected underlying interstitial lung disease (ILD). During that time, chest CT was done, which showed diffuse reticulonodular opacities with mediastinal and hilar lymphadenopathy. He also underwent bronchoscopy with extensive biopsies, which were negative for malignancy. He was discharged on a slow prednisone taper and 3 L oxygen. The patient was mildly hypertensive at 150/90 mmHg in the emergency department, while other vitals were within normal limits. A blood workup revealed a white blood cell count (WBC) of 8.4 g/dl, hemoglobin (Hb) of 16.5 g/dl, and platelet count of 208,000/mcL; however, he had lactic acid levels of 4.9 mmol/L, and blood urea nitrogen/creatinine (BUN/Cr) was 50/1.62. 

**Figure 1:**
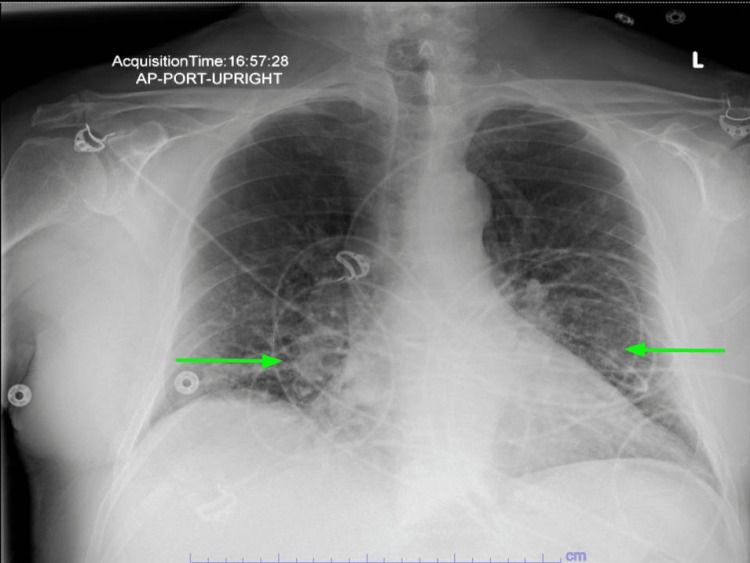
Chest x-ray showed streaky bibasilar airspace disease (green arrows).

**Figure 2:**
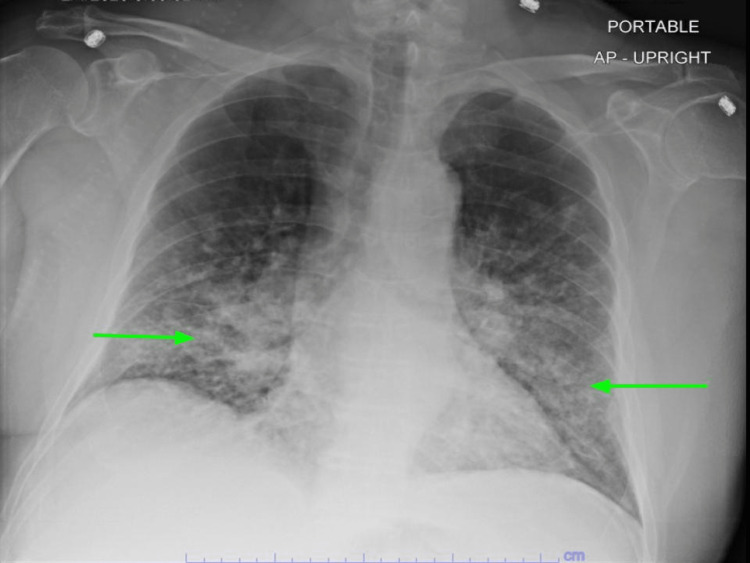
CXR showed increased patchy airspace disease throughout the mid- and lower-lung zones bilaterally (green arrows), suggesting multifocal pneumonia. CXR: Chest radiography

**Figure 3:**
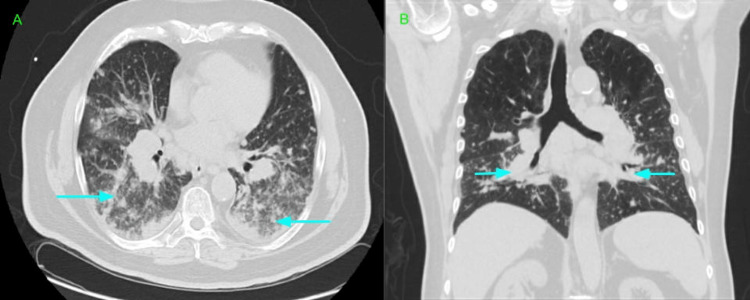
Chest CT without contrast (A: axial view; B: coronal view) revealed innumerable confluent nodules in both lungs with basilar predominance. There were ground-glass opacities in the lungs affected by the nodules (blue arrows).

**Figure 4:**
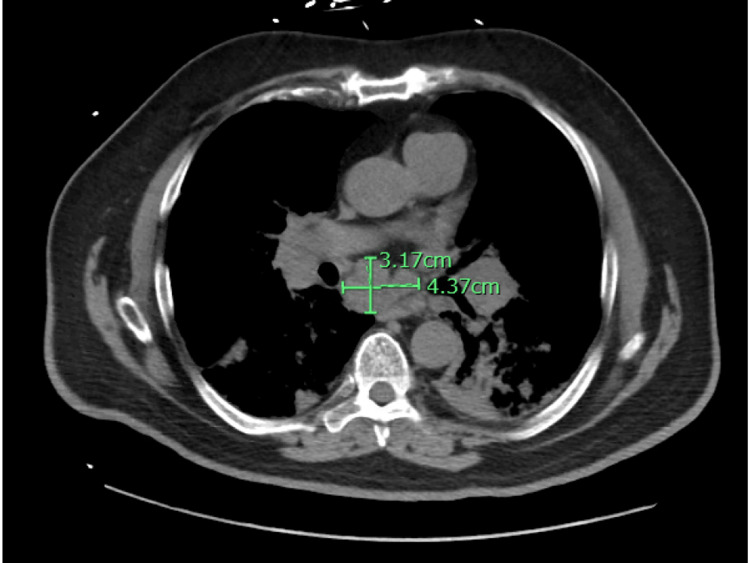
CT chest without contrast (axial view) showed extensive mediastinal and hilar adenopathy with conglomerate subcarinal lymph nodes measuring 4.37 x 3.17 cm.

**Figure 5:**
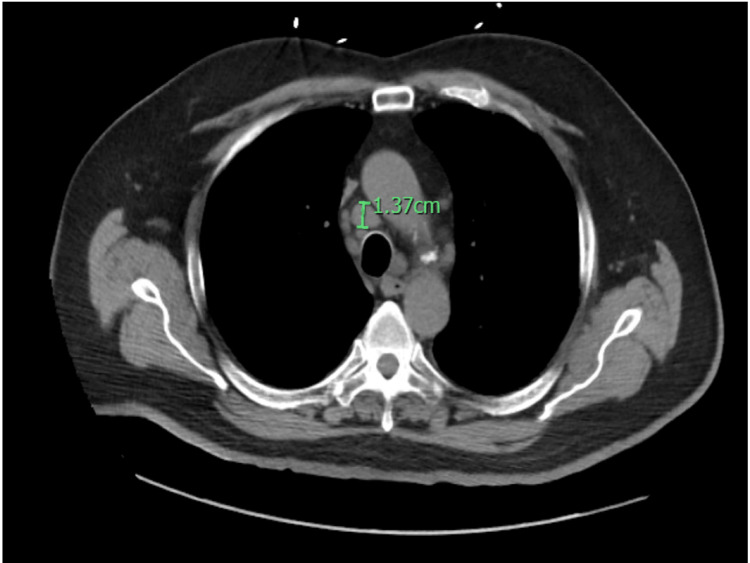
CT chest without contrast (axial view) showed the enlarged right peritracheal lymph node measuring 1.37 cm.

His alanine transaminase (ALT) level was 56 U/L, while his aspartate transaminase (AST) was at 63 U/L; both were mildly elevated. A chest x-ray showed the stable appearance of the previously described streaky bibasilar airspace disease (Figure 1). On the second day of admission, he developed shortness of breath with increasing oxygen requirements of up to 50 L/min of the heated high-flow nasal cannula oxygen. An arterial blood gas (ABG) analysis revealed the following results: a pH of 7.46, a pCO_2_ level of 30 mmHg, and a pO_2_ level of 69 mmHg. He was started on IV methyl prednisone, 40 mg every six hours, and empirical antibiotics with cefepime plus azithromycin. Following transfer to the progressive care unit (PCU), the patient was started on bilevel-positive airway pressure (BIPAP) therapy. Repeated chest radiography (CXR) showed increased patchy airspace disease throughout the mid- and lower-lung zones bilaterally, which was suggestive of multifocal pneumonia (Figure 2). Chest CT was done without contrast, which revealed innumerable confluent nodules in both lungs with basilar predominance. There were ground-glass opacities in the lungs affected by the nodules. These nodules appeared to contact the pleural surfaces and are perilymphatic, or less likely, randomly distributed nodules (Figure 3). There was extensive mediastinal and hilar adenopathy with conglomerate subcarinal lymph nodes measuring 4.4 x 3.2 cm (Figure 4). The enlarged right peritracheal lymph node measures 1.4 cm (Figure 5).

Further laboratory tests were conducted, including legionella urinary antigens, rheumatoid factor (RF), methicillin-resistant staph aureus (MRSA) screening, cytomegalovirus (CMV) serology, HIV, antineutrophil cytoplasmic antibodies (ANCAs), cytoplasmic-ANCA, perinuclear-ANCA, Anaplasma, Babesia polymerase chain reactions (PCRs), and COVID antinuclear capsid bodies were all negative. In addition, C3 and C4 complement levels, SARS-CoV-2 nuclear capsid antibodies, angiotensin-converting enzyme (ACE) levels, and serum protein electrophoresis (SPEP) with immunofixation were all within normal limits. An echocardiogram was done, which showed grade 1 diastolic dysfunction with a hyperdynamic left ventricle and an ejection fraction of 70-75%. The lower extremity (LE) duplex showed no deep vein thrombosis (DVT). The patient was transferred to the ICU to further manage progressive hypoxic respiratory distress of unclear etiology and repeat endobronchial ultrasound bronchoscopy (EBUS). He was admitted with a normal complete blood count (CBC), and later he developed leukocytosis (EBC of 32.9 × 10^9^/L with an absolute neutrophil count of 19,000/uL) as well as lymphocytosis (9,000/uL) and a slowly decreasing platelet count (44,000/mcL).

An open lung biopsy was then attempted; however, it was aborted as the patient became hemodynamically unstable, which required vasopressors after intubation with sedation. He was started on norepinephrine, phenylephrine, and vasopressin to maintain blood pressure. His lactic acid rose to 21 mmol/L, and he developed chronic hyperkalemia of around 5.5 mEq/L. Continuous renal replacement therapy (CRRT) was started. The Department of infectious diseases was consulted, and the patient’s antibiotic was broadened and changed to caspofungin and cefepime. Acyclovir was also started as he tested positive for Herpes simplex virus (HSV) type 1 with suspicion of HSV pneumonia. His blood and urine cultures were negative. He again underwent bronchoscopy, which showed patent bronchi. Broncho alveolar lavage (BAL) for silver staining for pneumocystis carinii pneumonia (PJP) returned negative. BAL fluid with gram staining showed no organisms. His pathology report showed no immunophenotypic evidence of a clonal B-cell population.

The oncology department was consulted, and a bone marrow biopsy was done as an open lung biopsy could not be obtained. His peripheral blood smear showed large atypical cells with a large round nucleus, slightly dispersed chromatin, prominent nucleoli, and basophilic cytoplasm. These atypical cells may represent reactive lymphocytes or monocytes. The flow cytometry was done, which found no evidence of lymphoproliferative disorder/abnormal myeloid maturation or increased blast population in peripheral blood. Unfortunately, the next day the patient had pulseless electrical activity (PEA) arrest, and cardiopulmonary resuscitation (CPR) was given, but the patient passed away on the 18th day of hospitalization.

**Figure 6:**
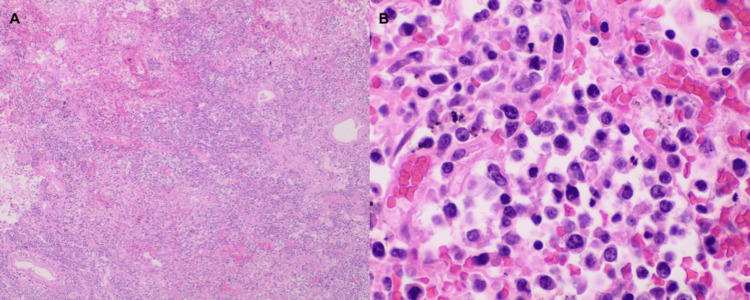
Sections of the lung showing diffuse effacement of the architecture by sheets of large malignant lymphoid cells that are positive for CD30 (A: H&E, x40; B: H&E, x400). H&E: Hematoxylin and eosin stain

**Figure 7:**
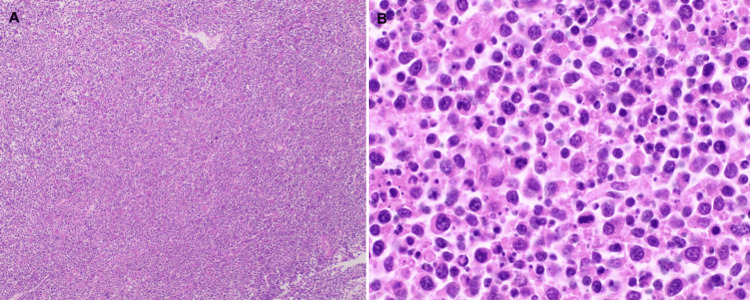
Sections of lymph nodes show effaced architecture with a diffuse infiltrate of neoplastic cells (A: H&E, x40). Neoplastic cells are pleomorphic and large, are composed of vesicular chromatin, have irregularly contoured nuclei, and possess prominent nucleoli. Mitotic figures and apoptotic bodies are seen. (B: H&E, x400). H&E: Hematoxylin and eosin stain

**Figure 8:**
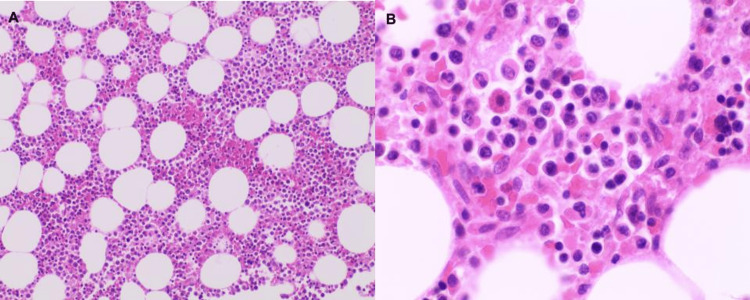
Sections of bone marrow showing mildly hypercellular marrow consisting of large atypical neoplastic lymphoid cells, present interstitially, and forming ill-defined aggregates that account for approximately 20% of total cellularity (A: H&E, x100). These large, atypical cells have curved and multilobed nuclei with golgi zone accentuation, known as hallmark cells (B: H&E, x400). H&E: Hematoxylin and eosin stain

**Figure 9:**
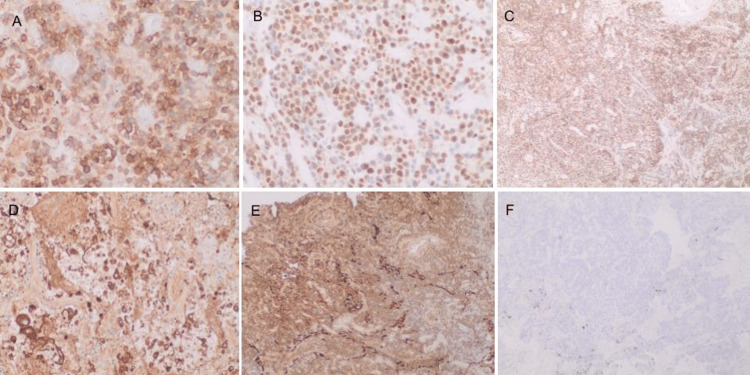
Immunohistochemical features of atypical lymphoid cells: A) Positive for CD30; B) positive for p63; C) positive for CD45/LCA; D) positive for EMA; E) positive for cytokeratin AE1/AE3; F) negative for ALK. EMA: Epithelial membrane antigen; ALK: Anaplastic lymphoma kinase; LCA: Leukocyte common antigen

The autopsy showed CD30-positive, ALK-negative ALCL diffusely involving all lung fields (Figure 6). The bronchioles, bronchi, and trachea were lined by a pink, congested mucosa, and the lumina were patent. Both the right and left pulmonary arteries were patent. Multiple paratracheal, paraaortic, bilateral hilar, bilateral peribronchial, and perirenal lymph nodes (0.5-4.1 cm) were diffuse infiltration by lymphoma cells consistent with CD30-positive, ALK-negative anaplastic large cells (Figure 7). Liver, bone marrow, and extracortical brain blood vessels were involved by CD30-positive, ALK-negative anaplastic large cells (Figure 8). By immunohistochemistry, these atypical lymphoid cells were positive for CD45 (leukocyte common antigen (LCA)), CD30, and p63. They were negative for ALK, epithelial membrane antigen (EMA), CD15, B-cell markers CD20 and PAX5, CD3, Granzyme, and epithelial markers cytokeratin AE1/AE3 and CAM 5.2 (Figure 9). The cause of death for this 72-year-old man was established to be CD30-positive, ALK-negative anaplastic large cell lymphoma involving numerous sites but especially the lungs, leading to respiratory compromise.

## Discussion

ALCL is a non-Hodgkin lymphoma and one of the subtypes of T-cell lymphoma that is positive for CD30 and is characterized by large, pleomorphic lymphoid cells with abundant cytoplasm and horseshoe-shaped nuclei [1]. The term anaplastic means that the lymphoma looks large and cancerous under the microscope. There are four major subtypes of ALCL as stated by the World health organization in 2016: Systemic, which includes ALK positive and negative, primary cutaneous and breast implant-associated ALCL [2]. Systemic ALCL is an aggressive CD30+ non-Hodgkin lymphoma. It commonly occurs in younger patients and includes both the ALK+ and ALK- variants. ALK-positive ALCL has a more favorable prognosis than ALK-negative ALCL with a five-year survival rate of 70%-90% [3]. While a cutaneous ALCL is one which commonly affects the skin and is characterized by red, itchy skin. Individuals diagnosed with ALK-negative ALCL are generally older (median age of diagnosis ranging from 54 to 61 years) compared to those with ALK-positive ALCL (median age of diagnosis is 27 years). The male-to-female ratio is similar between the ALK groups, with a ratio of 0.9 [4].

There is currently no specific risk factor that has been identified as a clear cause of ALCL. While some viruses have been known to cause NHL in humans, such as Epstein-Barr virus (EBV) and the human T-cell leukemia/lymphoma virus family, there is no convincing evidence that they play a role in the development of ALCL. In a recent study of 64 ALCL cases, no evidence of EBV was found. There is limited documentation of a correlation between ALCL and inherited immunological deficiency disease or other immunological disorders. However, recent research indicates that autoimmune disorders may contribute to the risk of the development of T-cell ALCL. Studies have shown that coeliac disease and psoriasis are associated with an increased risk of systemic T-cell ALCL, possibly due to chronic antigenic stimulation and local antigenic drive, ultimately leading to the development of lymphoma [5-6].

It’s extremely rare for systemic ALCL to initially present with acute respiratory failure. As per the case report series, there were nine cases of ALCL with respiratory failure [7]. Eight cases were of bronchial obstruction and one case was of tracheal obstruction. However, our case is an unusual one as the patient presented with a patent bronchus and trachea. It was only found in the autopsy that his lung parenchyma was diffusely involved with ALCL which slowly led to respiratory system dysfunction. Because ALCL is a rare type of cancer, studies evaluating new therapies often group ALCL with other peripheral T cell lymphomas (PTCL). Therefore, the current treatment approach for ALCL closely follows that of PTCL in general. Due to the high expression of CD30 in ALCL, the anti-CD30 antibody-drug conjugate, brentuximab vedotin, has been used to treat both systemic and primary cutaneous ALCL. Recent clinical trials with brentuximab have shown significant effectiveness in the treatment of relapsed or refractory ALCL. Currently, a randomized phase III study is underway to compare brentuximab plus CHP (cyclophosphamide, doxorubicin, prednisone) to standard CHOP (cyclophosphamide,doxorubicin, prednisone, vincristine) in the front-line setting [8]. Although relapses of ALCL (ALK-positive) can be treated with chemotherapy, allogeneic bone marrow transplantation is only recommended for refractory cases [9].

Our patient presented with a syncopal episode. During the course of his hospitalization, he developed progressive hypoxia requiring ICU admission. Later he progressed to acute respiratory failure with evolving pulmonary infiltrates despite the use of systemic steroids in the absence of fever. Furthermore, imaging reveals significant mediastinal and hilar lymphadenopathy creating a broad differential diagnosis. His post-endobronchial ultrasound assessment with transbronchial lymph node sampling revealed lymphoid tissue without any histopathologic findings. AFB sampling was negative via stain, and serum procalcitonin levels have been unremarkable. These constellations of findings argued against a bacterial lower respiratory tract infection at that point thus further investigation followed to find a suspected viral trigger. His intermittent steroid exposure could theoretically increase his risk for fungal infection, so he was tested for PJP but it turned out to be negative. He did not display characteristic features of HSV or Varicella zoster (VZV) pneumonia, and no mucocutaneous lesions were identified. The patient was also broadly investigated for autoimmune conditions but all work up came negative.

His bone marrow, EBUS, and BAL were non-diagnostic. The flow cytometry on lymph node biopsy and peripheral blood before this admission were also negative raising doubts about the lymphoproliferative disorder. His negative lymph node biopsy was suspected to be due to his being on high doses of steroids. Corticosteroids can be cytotoxic to lymphoma cells leading to false negative biopsy results [10,11]. Despite these findings, given significant mediastinal lymphadenopathy with continued progression of bilateral pulmonary infiltrates and lymphocytosis, and thrombocytopenia, we tried to get an open lung biopsy to see the entire lymph node architecture. But unfortunately, the procedure was abandoned as the patient's respiratory status deteriorated in the operation theater requiring vasopressors to maintain systolic blood pressure. This is a rare and interesting case of ALCL presented with acute respiratory failure without any bronchial or tracheal obstruction in imaging or bronchoscopy with negative bone marrow, and EBUS findings, which shows the clinical challenge posed for diagnosis. This case highlights the diagnostic challenge of ALCL presenting as rapidly progressive acute respiratory failure with negative lymph node biopsies. Despite extensive workup, the diagnosis was not made until after the patient's death. It emphasizes the importance of considering rare malignancies in the differential diagnosis and pursuing further diagnostic testing when the initial workup is inconclusive.

## Conclusions

Early diagnosis with lymph node biopsy is crucial for management and preventing life-threatening consequences of ALCL, but sometimes biopsies can be non-diagnostic when patients are on steroids. This case illustrates the rare presentation of ALCL posed diagnostic pitfall. Thus, rare malignancies should be considered for differential diagnoses when a diagnosis cannot be made based on an initial workup. At the same time, this will help the clinician to understand the uncommon entities of ALCL and maintain high suspicion when encountered.
